# Wave propagation, bi-directional reflectionless, and coherent perfect absorption-lasing in finite periodic PT-symmetric photonic systems

**DOI:** 10.1515/nanoph-2023-0157

**Published:** 2023-05-30

**Authors:** Jeng Yi Lee, Pai-Yen Chen

**Affiliations:** Department of Opto-Electronic Engineering, National Dong Hwa University, Hualien 974301, Taiwan; Department of Electrical and Computer Engineering, University of Illinois at Chicago, Chicago, IL 60661, USA

**Keywords:** bi-directional reflectionless, Bloch phase, coherent perfect absorption-lasing, finite periodic PT symmetric photonics, manipulation of wave scattering of PT symmetric photonics

## Abstract

With consideration of parity-time (PT) symmetry, Lorentz reciprocity theorem, and real Bloch phase, we propose a generalized parametric space for any PT-symmetric unit cells that can comprehensively illustrate the PT phase transition, Bloch phase, and necessary conditions of exotic wave scattering in the general finite periodic PT photonic structures. We put forward rigorous and formal expressions of bi-directional reflectionless and coherent perfect absorption and lasing (CPAL) for the finite one-dimensional PT photonic structures. With a new concept of the parametric space, we demonstrate the necessary PT phases of general unit cells, which result in the abnormal bi-directional reflectionless and CPAL effects. Moreover, thanks to parametrization, analytical formulas for complex relative permittivities of the unit cells composed of subwavelength gain–loss heterostructure are derived to provide a guideline for manipulating different PT scattering events. We accordingly study several one-dimensional PT photonic systems to achieve exotic wave scattering enabled by PT-symmetry. We believe this work may offer a theoretical underpinning for studying extraordinary wave phenomena of PT-symmetric photonics and may open avenues for manipulation of light.

## 1 Introduction

Inspired by an extension of quantum mechanics [1], non-Hermitian systems with parity-time (PT) symmetry have been studied in a variety of physical waves, such as photonics [2], acoustics [3–5], electric circuits [6–9], elastic plate [10, 11], coupled mechanical oscillators [12, 13] to name a few. Such systems characterized by a non-Hermitian Hamiltonian, violated by unitary relation, remain invariant under a combined PT operations.

Photonics with parity-time (PT) symmetry in one dimension would demand that the real part of refractive index in spatial placement has an even symmetry, while the imaginary part has an odd symmetry, corresponding to gain (amplification) and loss media (attenuation) embedded [14]. The corresponding scattering behaviors can be generally categorized into PT symmetry and broken symmetry phases. In between PT symmetry and broken symmetry phases, there exists an exceptional point with scattering eigenvalues and eigenstates coalesced, corresponding to an onset of symmetry-breaking transition [15]. It has experimentally demonstrated with coupled optical waveguides fabricated by Fe-doped LiNbO_3_ and AlGaAs [16, 17].

PT-symmetric systems operated at these distinctive PT phases can exhibit exotic scattering properties, including uni-directional invisibility [18–22], subdiffraction imaging [23, 24], coherent perfect absorber-laser [15, 25], PT-symmetric whispering gallery mode resonators [26, 27], uni-directional optical pulling force [28–30], superior sensing capability [7, 8, 31, 32], single-mode microrings lasing [33, 34], and Bloch oscillations [35].

Remarkably, these works have focused on a simple gain–loss architecture. Although there have been some efforts dedicated to periodic PT-symmetric structures of Refs. [18, 20, 36], [37], [38], [39], the relation of wave propagation and PT phase between unit cells and its finite periodic constructions in a more general way has not yet been explored.

In this work, we firstly propose a generalized parametric space obtained from consideration of PT-symmetric transfer matrix, Lorentz reciprocity theorem, and real Bloch phase. With this parametric space, we can not only point out a comprehensive relation between PT phase and Bloch phase for any unit cells, but also enable to discuss the formations of bidirectional reflectionless and coherent perfect absorption-lasing (CPAL) occurred at any finite periodic systems. Once systems are constituted by unit cells having an exceptional point or PT broken symmetry phase, it could enable wave propagation, independent of its transmission phase. However, as systems composed of unit cells operated at symmetry phase, the occurrence of wave propagation would depend on the transmission phase. With the help of the parametrization, we derive the general conditions for any unit cells to have bidirectional reflectionless and CPAL occurred at finite periodic systems. We find that by engineering proper PT phases and cell number, finite periodic systems can behave bi-directional reflectionless, even its unit cell is not operated at exceptional point. In addition, there are two solutions to achieve CPAL. One is to design unit cells having CPAL, while its cell number has to be odd. Another one is by designing a proper cell number and operating specific PT broken symmetry phase, as well as a null transmission phase of the unit cell. On the other hand, as unit cells made of gain–loss heterostructure with subwavelength scales, we derive the corresponding relative permittivity expressions to further manipulate wave scattering of assembly system. Consequently, we practically design several systems having these representative PT-symmetric scattering. We believe this work may provide an alternative method to realize extraordinary waves using a finite periodic structure.

## 2 Theory

### 2.1 Transfer matrix of finite periodic PT-symmetric systems

The transfer matrix of a unit cell is denoted as *M*_1_. Since the unit cell has a PT-symmetry, it obeys $M_{1}^{*}=M_{1}^{-1}$ , [25]. Then, we require that finite periodic structures composed of PT-symmetric unit cells are embedded in homogeneous environments. According to Lorentz reciprocity theorem, we have $Det\left[M_{1}^{N}\right]=1$ . Here *N* denotes total number of unit cells. Since $Det\left[M_{1}^{N}\right]=1$ is valid for any *N*, it would lead to *Det*[*M*_1_] = 1. Combined with $M_{1}^{*}=M_{1}^{-1}$ and *Det*[*M*_1_] = 1, we can obtain ${\left(M_{1}^{N}\right)}^{-1}={\left(M_{1}^{N}\right)}^{*}$ . The detailed analysis is placed in Appendix A. This result ensures that any N-cell structures composed by PT-symmetric unit cells still have a PT-symmetry invariant.

The transfer matrix for the unit cell having PT-symmetry can be parametrized by

(1) $$M_{1}=\left[\begin{array}{cc} \sqrt{1-\alpha_{1}\beta_{1}}{\text{e}}^{\text{i}\phi_{1}} & i\alpha_{1}\\ i\beta_{1} & \sqrt{1-\alpha_{1}\beta_{1}}{\text{e}}^{-\text{i}\phi_{1}} \end{array}\right]$$

here *α*_1_ and *β*_1_ are reals and *ϕ*_1_ is transmission phase of the unit cell bound by $\left[0,2\pi \right)$ , Refs [30, 40]. The exact description for *α*_1_, *β*_1_, and *ϕ*_1_ would depend on system configurations. Due to Lorentz reciprocity theorem established in the unit cell, i.e., *Det*[*M*_1_] = 1, there has a constraint for *α*_1_ and *β*_1_, i.e., *α*_1_ *β*_1_ ≤ 1 [30, 40].

Next, for our PT-symmetric systems composed by N PT-symmetric unit cells, with the help of Chebyshev identity, the resultant transfer matrix reads

(2) $$\begin{array}{cc} M_{1}^{N} & ={\left[\begin{array}{cc} \sqrt{1-\alpha_{1}\beta_{1}}{\text{e}}^{\text{i}\phi_{1}} & i\alpha_{1}\\ i\beta_{1} & \sqrt{1-\alpha_{1}\beta_{1}}{\text{e}}^{-\text{i}\phi_{1}} \end{array}\right]}^{N}\\ & =\left[\begin{array}{cc} \sqrt{1-\alpha_{1}\beta_{1}}{\text{e}}^{\text{i}\phi_{1}}U_{N-1}-U_{N-2} & i\alpha_{1}U_{N-1}\\ i\beta_{1}U_{N-1} & \sqrt{1-\alpha_{1}\beta_{1}}{\text{e}}^{-\text{i}\phi_{1}}U_{N-1}-U_{N-2} \end{array}\right]\\ & =\left[\begin{array}{cc} \frac{1}{t_{N}^{*}} & \frac{r_{R,N}}{t_{N}}\\ -\frac{r_{L,N}}{t_{N}} & \frac{1}{t_{N}} \end{array}\right] \end{array}$$

where *t*_*N*_ is transmission coefficient, *r*_*R*,*N*_ is right reflection coefficient, *r*_*L*,*N*_ is left reflection coefficient, $U_{N}=\frac{\sin\left[\left(N+1\right)\Phi \right]}{\sin\Phi }$ , and Φ is Bloch phase [36, 37, 41]. The Bloch phase is related to the eigenvalues (*λ*_1_ and *λ*_2_) of *M*_1_,

(3) $$\cos\Phi =\frac{1}{2}\left(\lambda_{1}+\lambda_{2}\right)=\frac{1}{2}\mathrm{Tr}\left[M_{1}\right]=\sqrt{1-\alpha_{1}\beta_{1}}\cos\left[\phi_{1}\right].$$

The condition of Im[Φ] = 0 enables incident waves to propagate through assembly systems.

### 2.2 3D generalized parametric space for general PT-symmetric unit cells enabling wave propagation

**Figure 1:**
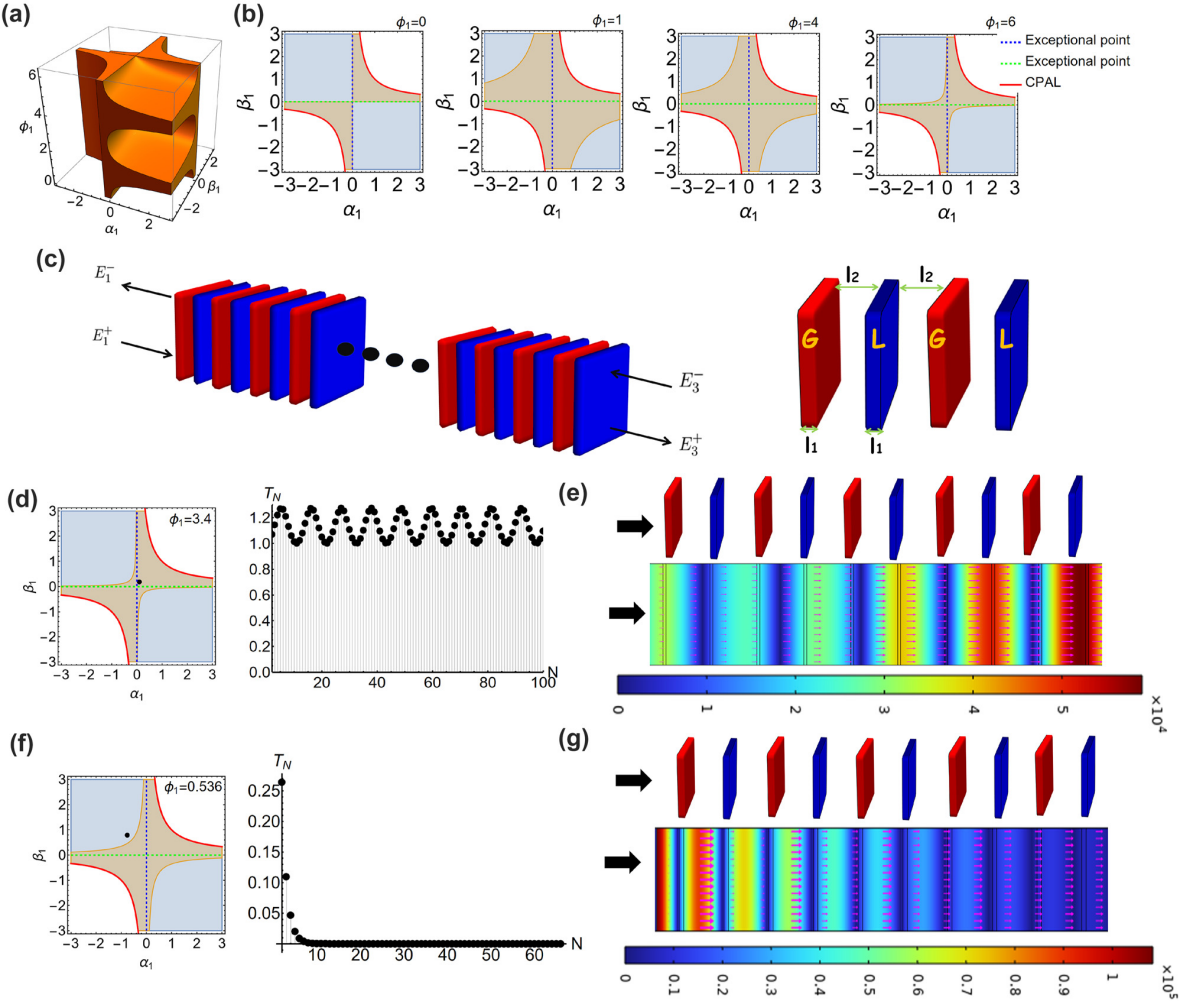
With consideration of PT-symmetry, Lorentz reciprocity theorem, and real Bloch phase, we depict the 3D parametric space in terms of parametrization *α*_1_, *β*_1_, and *ϕ*_1_ for any PT-symmetric unit cells supporting wave propagation in (a). More specifically, by taking transmission phases of unit cells by *ϕ*_1_ = 0, 1, 4, 6, we provide the corresponding 2D parametric space by *α*_1_ and *β*_1_ marked by light-orange colors in (b). The dark-blue regions denote the accessible parametrization for general PT-symmetry systems, while it possesses complex Bloch phase, resulting in forming evanescent waves in periodic systems. Here red-solid, green-dashed, and blue-dashed lines denote as CPAL, exceptional point and exceptional point, respectively. (c) Schematic of finite periodic PT-symmetric photonics made of a PT-symmetric unit cell with simple gain (G)–loss (L) heterostructure. Here the gain slabs are depicted by red colors, while the loss are depicted by blue colors. The refractive index of the gain (loss) slab is denoted as *n*_*g*_ $\left(n_{l}=n_{g}^{*}\right)$ . $E_{1}^{+}$ and $E_{1}^{-}$ ( $E_{3}^{+}$ and $E_{3}^{-}$ ) are complex amplitudes of electromagnetic plane waves in the left lead (right lead) toward right and left propagation, respectively. We illustrate the corresponding parametrization from two sets of unit cells in the parametric space marked by block dots as shown in the left panel of (d)–(f), while the corresponding transmittances *T*_*N*_ with *N* are shown in the right panel of (d)–(f), respectively. With commercial software COMSOL, we plot the instantaneous electric field distribution and time-averaged Poynting vectors (pink arrows) for the corresponding systems composed by 5 unit cells in (e)–(g). In the case of (d)–(e), the parameters for the unit cell are *n*_*l*_ = 1.82 + 0.38*i*, *l*_1_ = 0.0159*λ*_0_ and *l*_2_ = 0.237*λ*_0_, while in the case of (f)–(g) the parameters are *n*_*l*_ = 1.92 + 0.134*i*, *l*_1_ = 0.048*λ*_0_ and *l*_2_ = 0.442*λ*_0_. Here *λ*_0_ is operating wavelength.

Equation (3) indicates that the Bloch phase depends on the PT phase and transmission phase of the unit cells, i.e., *α*_1_, *β*_1_, and *ϕ*_1_. Accordingly, with PT-symmetry, Lorentz reciprocity theorem, and real Bloch phases (Im[Φ] = 0), we provide a 3D parametric space in terms of *α*_1_, *β*_1_, and *ϕ*_1_, to display allowable parametrization supporting wave propagation in finite periodic PT-symmetric structures, marked by light-orange colors in Figure 1(a).

More specifically, we plot 2D parametric spaces by taking *ϕ*_1_ = 0, 1, 4, 6, where the real Bloch phases are depicted by light-orange colors in Figure 1(b). For a comparison, we also depict the accessible parametrization for general PT-symmetric systems marked by dark-blue regions. We can see that not every PT accessible regions can support wave propagation in finite periodic systems. In the PT broken symmetry phase and exceptional point, it requires 0 < *α*_1_ *β*_1_ ≤ 1 and *α*_1_ *β*_1_ = 0 by parametrization, respectively. These conditions would lead to have real Bloch phase, since it follows $0\leq \sqrt{1-\alpha_{1}\beta_{1}}\leq 1$ . Thus, any PT-symmetric unit cells operated at the PT broken symmetry phase or exceptional point would ensure wave propagation in any N-cell systems. We also note that in this case, the transmission phase of the unit cell can be arbitrary. On the other hand, we can observe that some regions of PT symmetry phase have complex Bloch phase, as indicated by dark-blue colors in Figure 1(b), where depend on *ϕ*_1_. Since the symmetry phase corresponds to *α*_1_ *β*_1_ < 0 by parametrization, the factor of Eq. (3) would be subject to $\sqrt{1-\alpha_{1}\beta_{1}}>1$ by some values of *α*_1_ *β*_1_, leading to the emergence of complex Bloch phase.

## 3 Design of PT-symmetric unit cells by parametrization

We emphasize that the aforementioned discussion goes beyond any specific system configurations, materials, and operating frequency. On the other hand, with the state-of-art development of metasurface, the dimension of the system is toward deep subwavelength. From the practical perspective, we discuss a PT-symmetric periodic photonic system made by a unit cell with simple gain–loss heterostructure having deep subwavelength scales, as shown in Figure 1(c). The refractive indexes for gain and loss are denoted as *n*_*g*_ and $n_{l}=n_{g}^{*}$ , respectively, and the thickness for each slabs is denoted as *l*_1_. The distance between two adjacent slabs is *l*_2_. In between slabs, there is an air gap. Under the condition of deep subwavelength dimension for the slab inclusions, we can obtain the corresponding complex relative permittivities,

(4) $$\begin{array}{ll} \epsilon_{l}^{\prime \prime } & =\frac{e^{ik_{0}l_{2}}\left(\alpha_{1}+\beta_{1}\right)}{k_{0}l_{1}\left[i-ie^{2ik_{0}l_{2}}+k_{0}l_{1}+k_{0}l_{1}e^{2ik_{0}l_{1}}\right]},\\ \epsilon_{l}^{\prime \pm } & =\frac{e^{ik_{0}l_{2}}}{k_{0}^{2}l_{1}^{2}\left(-1+e^{2ik_{0}l_{2}}\right)}\left(2ik_{0}l_{1}\cos\left[k_{0}l_{2}\right]\right.\\ & \;\pm \left.\sqrt{-2k_{0}^{2}l_{1}^{2}\left[1-k_{0}^{2}l_{1}^{2}\left(-1+\epsilon_{l}^{\prime \prime 2}\right)+\left(1+k_{0}^{2}l_{1}^{2}\left(-1+\epsilon_{l}^{\prime \prime 2}\right)\right)\cos\left(2k_{0}l_{2}\right)+2\left(-\alpha_{1}+\beta_{1}\right)\sin\left(k_{0}l_{2}\right)-2k_{0}l_{1}\sin\left(2k_{0}l_{2}\right)\right]}\right), \end{array}$$

here $n_{l}^{2}=\epsilon_{l}^{\prime }+i\epsilon_{l}^{\prime \prime }$ and $n_{g}^{2}=n_{l}^{*2}=\epsilon_{l}^{\prime }-i\epsilon_{l}^{\prime \prime }$ , operating wavenumber of *k*_0_ = 2π/*λ*_0_, and operating wavelength of *λ*_0_. The corresponding transmission phase of the unit cell reads

(5) $$\phi_{1}=Arg\left[\frac{4e^{2ik_{0}l_{2}}}{4+k_{0}l_{1}\left[-2i\left(2+2\epsilon_{l}^{\prime \pm }\right)+k_{0}l_{1}\left(e^{2ik_{0}l_{2}}\left(-1+\epsilon_{l}^{\prime \pm }-i\epsilon_{l}^{\prime \prime }\right)\left(-1+\epsilon_{l}^{\prime \pm }+i\epsilon_{l}^{\prime \prime }\right)-\left(1+\epsilon_{l}^{\prime \pm }-i\epsilon_{l}^{\prime \prime }\right)\left(1+\epsilon_{l}^{\prime \pm }+i\epsilon_{l}^{\prime \prime }\right)\right)\right]}\right]$$

The detailed derivation is placed in the Appendix B.

To have gain materials, there can be conventionally achieved by electric pumping or optical pumping. For the former, by adjusting electric carrier injection in III–V semiconductors, one can modulate the gain parameters. For the latter, it can be nanostructured by quantum dots, quantum wells, dye molecules, halide perovskites and graphene metasurface [31, 42].

## 4 Numerical results and discussion

### 4.1 Propagating wave

By taking parametrization (*α*_1_ = 0.1 and *β*_1_ = 0.174) and geometry (*l*_1_ = 0.0159*λ*_0_ and *l*_2_ = 0.237*λ*_0_) into Eqs. (4) and (5), we can obtain the corresponding complex permittivities for the loss slab of *n*_*l*_ = 1.82 + 0.38*i* and the transmission phase of *ϕ*_1_ = 3.4. This design of the unit cell correspond to a real Bloch phase and broken symmetry phase, whose its parametrization (*α*_1_ = 0.1 and *β*_1_ = 0.174) is marked by a black dot in the left panel of Figure 1(d). We calculate the resultant transmittance *T*_*N*_ = |*t*_*n*_|^2^ with *N* and plot the instantaneous electric field distribution and time-averaged Poynting vectors for the system with 5-cells in the right panel of Figure 1(d) and (e), respectively, enabling the wave propagation. Next, we choose another parametrization by (*α*_1_ = −0.76 and *β*_1_ = 0.78) and geometry parameters by (*l*_1_ = 0.048*λ*_0_ and *l*_2_ = 0.442*λ*_0_). By Eqs. (4) and (5), we obtain *n*_*l*_ = 1.92 + 0.134*i* and the transmission phase of *ϕ*_1_ = 0.536. In this design, the unit cell is operated at PT symmetry phase and has a complex Bloch phase, whose its parametrization is marked by a block dot in the left panel of Figure 1(f). As expected, wave propagation in the periodic system constituted by such unit cell would form an evanescent wave as shown in the right panel of Figure 1(f), also supported by the instantaneous electric field and time-averaged Poynting vectors in Figure 1(g).

### 4.2 Bi-directional reflectionless

Any PT-symmetric systems operated at an exceptional point would accompany by unidirectional or bidirectional reflectionless, as well as unity transmittance [30, 37, 40, 43]. Since the transmission phase of PT-symmetric system operated at an expectational point can be arbitrary, the exotic scattering phenomenon can not be regarded as rigorous invisibility [43]. We note that the delay time, defined as the reflection coefficient with respect to operating frequency, behaves like a delta function at an exceptional point, due to an absence of reflected wave [44]. This leads to one-way (unidirectional transparency) or two-way (bi-directional transparency) light-trapping resonance in PT-symmetric systems, which the wave would be trapped for a long time until complete absorption by lossy materials. Moreover, the bifurcation of scattering eigenvectors near the exceptional point exhibits the potential applications for designs of enhanced photonic sensors [8, 45]. Recent studies further unveil that the emergence of the exceptional point is not a signature of PT-symmetric systems, while it is associated with non-Hermitian systems [2, 45, 46].

Any PT-symmetric unit cells at an exceptional point follow ${\left(M_{1}\right)}_{12}=0$ or ${\left(M_{1}\right)}_{21}=0$ or ${\left(M_{1}\right)}_{12}={\left(M_{1}\right)}_{21}=0$ , corresponding to *α*_1_ = 0 or *β*_1_ = 0 or *α*_1_ = *β*_1_ = 0 in parameterization. With these conditions, we can see that the wave scattering of systems made of the unit cells would also operate at an exceptional point. However, another condition to have an exceptional point in finite periodic systems, in the absence of the unit cell operated at the exceptional point, is

(6) sin[NΦ]=0,

already proposed by Refs. [37, 38]. We note that this condition would exhibit the bidirectional reflectionless, because it results in ${\left(M_{1}^{N}\right)}_{12}=0$ and ${\left(M_{1}^{N}\right)}_{21}=0$ (*r*_*R*,*N*_ = 0 and *r*_*L*,*N*_ = 0) simultaneously.

**Figure 2:**
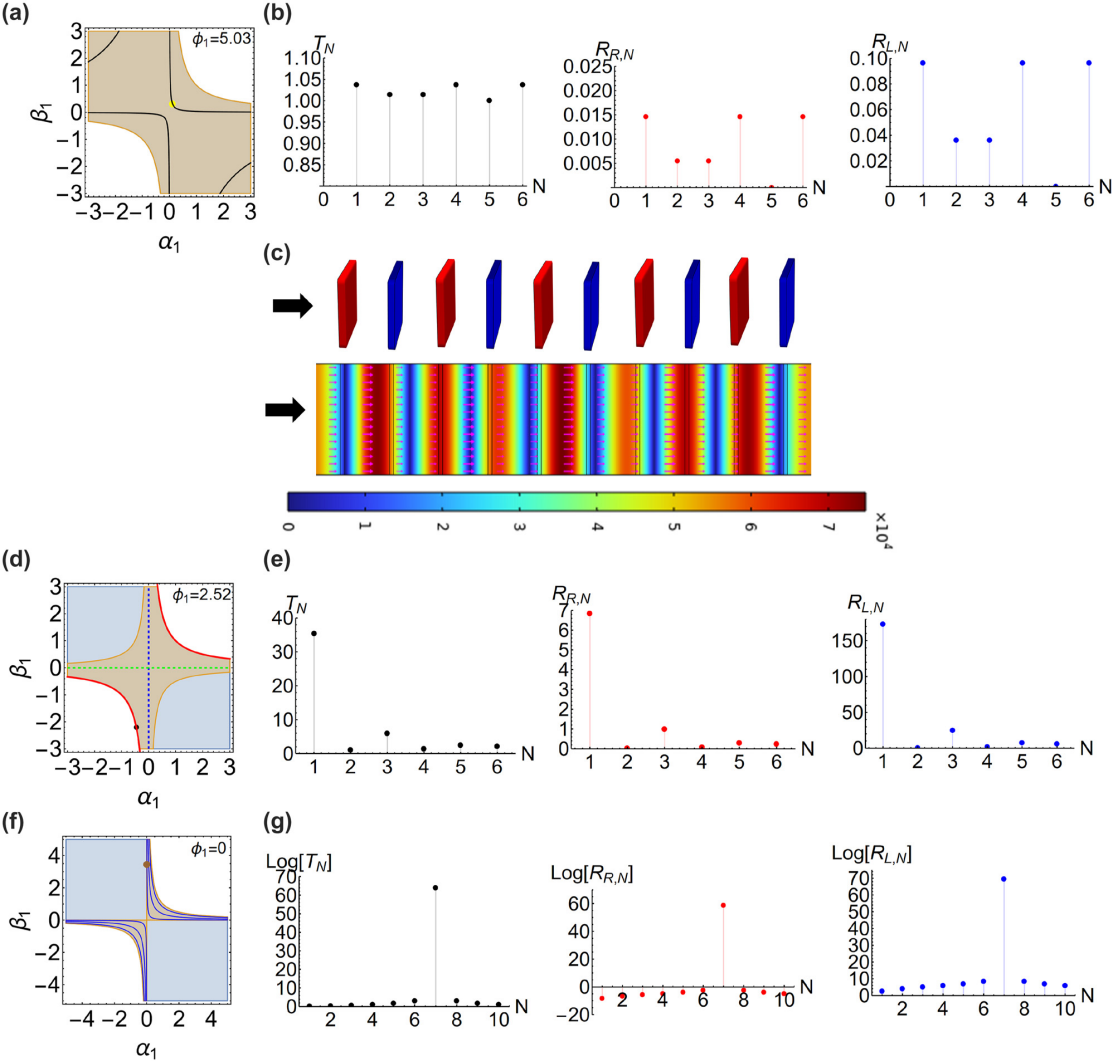
In (a), we depict the numerical solutions of Eq. (6) with *N* = 5 by black lines in the parametric space with *ϕ*_1_ = 5.03. With the design of *n*_*l*_ = 1.33 + 0.64*i*, *l*_1_ = 0.03*λ*_0_, and *l*_2_ = 0.36*λ*_0_, we mark the corresponding parametrization by a yellow dot. In this design, the system constituted of the 5-cells exhibits bi-directional reflectionless, as in (b). With commercial software COMSOL, we plot the corresponding instantaneous electric field distribution and time-averaged Poynting vectors (pink arrows) in (c). In (d), we design another unit cell operated at CPAL by using *n*_1_ = 1.34 + 1.09*i*, *l*_1_ = 0.07*λ*_0_, and *l*_2_ = 0.67*λ*_0_, whose the parametrization is marked by a black dot in the parametric space. The transmission phase is *ϕ*_1_ = 2.52. In (e), we calculate *T*_*N*_, *R*_*R*,*N*_, and *R*_*L*,*N*_ with *N*. In (f), we depict the numerical solutions of Eq. (8) with *N* = 7 in the parametric space marked by blue lines. We design another unit cell by using *n*_1_ = 3.17 + 3.14*i*, *l*_1_ = 0.016*λ*_0_, and *l*_2_ = 0.32*λ*_0_, that the parametrization is depicted by a brown dot. In (g), as expected, when at *N* = 7, the wave scattering of the system exhibits CPAL.

To demonstrate this result, we numerically solve sin[*N*Φ] = 0 with *N* = 5 and depict the solutions in the parametric phase marked by the black lines in Figure 2(a). The solutions correspond to the symmetry phase and broken symmetry phase, consistent with transmittance relation [47]. Now, we choose parametrization (*α*_1_, *β*_1_) = (0.12, 0.3) for our design, which meets the solution of Eq. (6) with *N* = 5, marked by a yellow dot in Figure 2(a). Then, taking geometry parameters (*l*_1_ = 0.03*λ*_0_ and *l*_2_ = 0.36*λ*_0_) into Eqs. (4) and (5), we obtain *n*_*l*_ = 1.33 + 0.64*i* and the transmission phase of *ϕ*_1_ = 5.03. Here the unit cell is at broken symmetry phase and has real Bloch phase. In Figure 2(b), we calculate the corresponding transmittance *T*_*N*_, left reflectance *R*_*L*,*N*_ = |*r*_*L*,*N*_|^2^, and right reflectance *R*_*R*,*N*_ = |*r*_*R*,*N*_|^2^ with *N*. We can see that at *N* = 5, the assembly system reflects *T*_*N*_ = 1 and *R*_*L*,*N*_ = *R*_*R*,*N*_ = 0, also supported by the plot of instantaneous electric field distribution and Poynting vectors in Figure 2(c).

### 4.3 Coherent perfect absorption-lasing (CPAL)

Any PT-symmetric systems operated at CPAL have zero and infinity (pole) eigenvalues of the scattering matrix, corresponding to coherent perfect absorption and lasing in wave scattering [15, 25]. The CPAL is a unique feature and discrete point of the PT broken symmetry phase. To have CPAL occurred at N-cell systems, it requires ${\left(M_{1}^{N}\right)}_{11}={\left(M_{1}^{N}\right)}_{22}=0$ and $Im\left[{\left(M_{1}^{N}\right)}_{12}\right]Im\left[{\left(M_{1}^{N}\right)}_{21}\right]=1$ . Accordingly, there are two solutions. (i) the unit cell exhibits CPAL and the corresponding cell number is odd. By parametrization, we read

(7) $$\left\{\begin{array}{c} \begin{array}{cc} \alpha_{1}\beta_{1} & =1\\ N & =1,3,5,. \end{array}\; \end{array}\right.$$

To our knowledge, this result is not found in other works.

(ii) The unit cell is not at CPAL, while its transmission phase is null and the Bloch phase should meet cos[*N*Φ] = 0,

(8) $$\left\{\begin{array}{c} \begin{array}{cc} \phi_{1} & =0\\ \cos\left[N\Phi \right] & =0. \end{array}\; \end{array}\right.$$

The detailed derivation is placed at Appendix C.

To verify the case (i), with Eqs. (4) and (7), we design the unit cell by *n*_1_ = 1.34 + 1.09*i*, *l*_1_ = 0.07*λ*_0_, and *l*_2_ = 0.67*λ*_0_, marked by a black dot in Figure 2(d), with CPAL property. The corresponding transmission phase for this unit cell is *ϕ*_1_ = 2.52. In Figure 2(e), we calculate *T*_*N*_, *R*_*R*,*N*_, and *R*_*L*,*N*_, with *N*. When *N* is odd, the system exhibits CPAL.

To verify the case (ii), we solve cos[*N*Φ] = 0 with *N* = 7 illustrated by the blue lines, corresponding to CPAL occurred at assembly 7-cell systems in Figure 2(f). We can observe that these lines are found at broken symmetry phases, consistent with transmittance relation, [47]. Then, taking the parametrization (*α*_1_, *β*_1_) = (0.014, 3.43*λ*_0_) obtained from the numerical solution of Eq. (8) and geometry parameters (*l*_1_ = 0.016*λ*_0_ and *l*_2_ = 0.32*λ*_0_) into Eq. (4), we obtain *n*_1_ = 3.17 + 3.14*i*. We mark this parametrization in the parametric space by a brown dot in Figure 2(f). The corresponding transmission phase of this unit cell is exactly null, *ϕ*_1_ = 0. Although this unit cell is not operated at CPAL, at *N* = 7, the system turns to behave CPAL, as shown in Figure 2(g).

**Figure 3:**
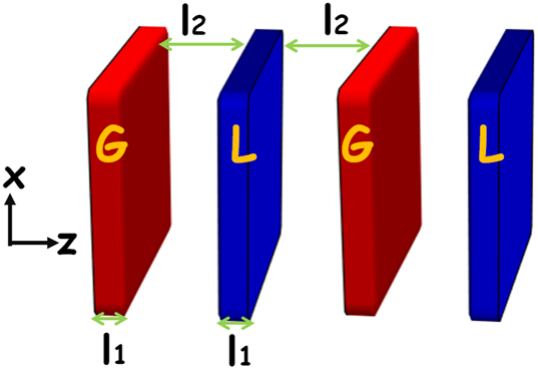
Schematic of a unit cell made of simple gain (G)-gap-loss (L) heterostructure. Here the refractive indexes for the gain and loss are *n*_*g*_ and $n_{l}=n_{g}^{*}$ , respectively, and the thickness of these slabs is denoted as *l*_1_. In between gain and loss, it is an air gap with a distance of *l*_2_ between adjacent gain and loss.

Besides the design of the unit cells by simple gain–loss configuration, the inverse calculation demonstrated here can be extended into more complicated designs of unit cells. Last but not least, we emphasize that the use of the parametric space here is not only directly from the consideration of PT-symmetric transfer matrix and Lorentz reciprocity theorem, but also from extended consideration of real Bloch phase. Therefore it could provide the comprehensive relation for PT phase and Bloch phase of general unit cells to construct exotic PT-symmetric wave phenomena in finite periodic systems.

## 5 Conclusions

Considering the Lorentz reciprocity theorem, PT-symmetry condition, and real Bloch phase, we have derived and exploited the 3D generalized parametric space to analyze the scattering behavior of finite periodic PT-symmetric structures. The parametric space provides the comprehensive relation between PT phase and Bloch phase for any PT-symmetric unit cells, beyond specific system configurations, materials, and operating frequency. Any PT synthetic electromagnetic systems composed of unit cells having broken symmetry phase and exceptional point would enable wave propagation. Interestingly, once any unit cell is operated at PT-symmetry phase, wave propagation in its constructed finite periodic systems would depend on its transmission phase. With parametrization, we derive the conditions for bi-directional reflectionless and CPAL occurred at N-cell systems. We comprehensively illustrate a variety of PT phases for the unit cells found by using the parametric space to achieve bi-directional reflectionless or CPAL in general N-cell systems. With parametrization, parametric space and analytical formulas for complex relative permittivities, we design several systems constituted by simple gain–loss heterostructure having deep subwavelength dimension to have representative PT scattering events. By means of the generalized parametric space, it can provide an universally applicable guideline for design and implementation for other PT-symmetric wave systems.

## Appendix A

For a unit cell with PT-symmetry, we have *M* ^−1^ = *M**, [25]. On the other hand, once the system has same opposite leads, we can have *Det*[*M* ^*N*^] = 1 from Lorentz reciprocity theorem.

In the following analysis, we would infer *Det*[*M*] = 1 and ${\left(M^{N}\right)}^{-1}={\left(M^{N}\right)}^{*}$ , based on two given conditions *M* ^−1^ = *M** and *Det*[*M* ^*N*^] = 1.

Since *Det*[*M* ^*N*^] = 1 is valid for any *N*, we argue

(10)
$$\begin{array}{cc} Det\left[M^{N}\right]=Det\left[M\right]Det\left[M\right].Det\left[M\right] & =1\\ ∴Det\left[M\right] & =1. \end{array}$$

Consider *AAA* ^−1^ *A* ^−1^ = *I* = (*AA*)(*A* ^−1^ *A* ^−1^) = *A* ^2^ *B* where *A* is invertible matrix and *I* is unit matrix. From $B={\left(A^{2}\right)}^{-1}$ , we can find $B={\left(A^{-1}\right)}^{2}={\left(A^{2}\right)}^{-1}$ . Thus, $M^{-1}M^{-1}\ldots M^{-1}={\left(M^{-1}\right)}^{N}={\left(M^{N}\right)}^{-1}$ . Using PT symmetry condition, i.e., *M* ^−1^ = *M**, we have

(11)
$$\begin{array}{cc} {\left(M^{-1}\right)}^{N} & ={\left(M^{N}\right)}^{-1}\\ ∴{\left(M^{*}\right)}^{N} & ={\left(M^{N}\right)}^{*}={\left(M^{N}\right)}^{-1}. \end{array}$$

Thus we obtain ${\left(M^{N}\right)}^{*}={\left(M^{N}\right)}^{-1}$ .

## Appendix B

The input electric fields, denoted as ${\vec{E}}_{1}^{+}$ and ${\vec{E}}_{2}^{-}$ , are *x*-polarized in our considered system in Figure 3. The time evolution of the electromagnetic fields is e^−i*ωt*^ where *ω* is operating angular frequency. The unit cell is constituted by simple gain-gap-loss heterostructure. The gap is air with a distance between adjacent gain/loss of *l*_2_. The refractive indexes for the gain and loss are *n*_*g*_ and $n_{l}=n_{g}^{*}$ , respectively. The transfer matrix for a slab with refractive index of *n* and thickness of *l*_1_ is

(12)
$$M_{\mathrm{slab}}\left(nk_{0},l_{1}\right)=\left[\begin{array}{cc} \cos\left[nk_{0}l_{1}\right]+\frac{i}{2}\sin\left[nk_{0}l_{1}\right]\left[\frac{1}{n}+n\right] & \frac{i}{2}\sin\left[nk_{0}l_{1}\right]\left[n-\frac{1}{n}\right]\\ -\frac{i}{2}\sin\left[nk_{0}l_{1}\right]\left[n-\frac{1}{n}\right] & \cos\left[nk_{0}l_{1}\right]-\frac{i}{2}\sin\left[nk_{0}l_{1}\right]\left[\frac{1}{n}+n\right] \end{array}\right]$$

[41], here *k*_0_ = 2π/*λ*_0_ is operating wavenumber. For the unit cell shown in Figure 3, the corresponding transfer matrix is

(13)
$$\begin{array}{ll} \left[\begin{array}{c} E_{3}^{+}\\ E_{3}^{-} \end{array}\right] & =M_{t}\left(\frac{l_{2}}{2}\right)M_{\mathrm{slab}}\left(n_{l}k_{1},l_{1}\right)M_{t}\left(l_{2}\right)M_{\mathrm{slab}}\left(n_{g}k_{1},l_{1}\right)M_{t}\left(\frac{l_{2}}{2}\right)\left[\begin{array}{c} E_{1}^{+}\\ E_{1}^{-} \end{array}\right]=M_{\mathrm{tot}}\left[\begin{array}{c} E_{1}^{+}\\ E_{1}^{-} \end{array}\right]. \end{array}$$

Here

(14)
$$M_{t}\left(l_{2}\right)=\left[\begin{array}{cc} e^{ik_{0}l_{2}} & 0\\ 0 & e^{-ik_{0}l_{2}} \end{array}\right].$$

By using the condition of deep wavelength scale for the gain and loss, we approximate sin[*nk*_0_ *l*_1_] ≈ *nk*_0_ *l*_1_ and cos[*nk*_0_ *l*_1_] ≈ 1. Thus, we obtain

(15)
$$\begin{array}{ll} \epsilon_{l}^{\prime \prime } & =\frac{e^{ik_{0}l_{2}}\left(\alpha +\beta \right)}{k_{0}l_{1}\left[i-ie^{2ik_{0}l_{2}}+k_{0}l_{1}+k_{0}l_{1}e^{2ik_{0}l_{1}}\right]}\\ \epsilon_{l}^{\prime } & =\frac{e^{ik_{0}l_{2}}}{k_{0}^{2}l_{1}^{2}\left(-1+e^{2ik_{0}l_{2}}\right)}\left(2ik_{0}l_{1}\cos\left[k_{0}l_{2}\right]\right.\\ & \;\pm \left.\sqrt{2}\sqrt{-k_{0}^{2}l_{1}^{2}\left[1-k_{0}^{2}l_{1}^{2}\left(-1+\epsilon_{l}^{\prime \prime 2}\right)+\left(1+k_{0}^{2}l_{1}^{2}\left(-1+\epsilon_{l}^{\prime \prime 2}\right)\right)\cos\left(2k_{0}l_{2}\right)+2\left(-\alpha +\beta \right)\sin\left(k_{0}l_{2}\right)-2k_{0}l_{1}\sin\left(2k_{0}l_{2}\right)\right]}\right). \end{array}$$

The transmission phase could be further derived

(16)
$$\phi_{1}=Arg\left[\frac{4e^{2ik_{0}l_{2}}}{4+k_{0}l_{1}\left[-2i\left(2+2\epsilon_{l}^{\prime }\right)+k_{0}l_{1}\left(e^{2ik_{0}l_{2}}\left(-1+\epsilon_{l}^{\prime }-i\epsilon_{l}^{\prime \prime }\right)\left(-1+\epsilon_{l}^{\prime }+i\epsilon_{l}^{\prime \prime }\right)-\left(1+\epsilon_{l}^{\prime }-i\epsilon_{l}^{\prime \prime }\right)\left(1+\epsilon_{l}^{\prime }+i\epsilon_{l}^{\prime \prime }\right)\right)\right]}\right]$$

## Appendix C

To occur CPAL at periodic PT-symmetric systems, the necessary conditions are ${\left(M_{1}^{N}\right)}_{11}={\left(M_{1}^{N}\right)}_{22}=0$ and $Im\left[{\left(M_{1}^{N}\right)}_{12}\right]Im\left[{\left(M_{1}^{N}\right)}_{21}\right]=1$ . There are two solutions.

(i) As the unit cell is operated at CPAL, i.e., *α*_1_ *β*_1_ = 1, the remaining conditions are *U*_*N*−2_ = 0 and ${\left(U_{N-1}\right)}^{2}=1$ . Therefore,
  (17)
  sin[(N−1)Φ]=0
  and
  (18)
  sin[NΦ]=±1.
  To simultaneously satisfy above equations, we need to require Φ = π/2, 3π/2 and odd *N*, i.e., *N* = 1, 3, 5, 7, …
  (ii) If the unit cell is not operated at CPAL, i.e., *α*_1_ *β*_1_ ≠ 1, we need
    (19)
    $$\begin{array}{cc} Im\left[{\left(M_{1}^{N}\right)}_{12}\right]Im\left[{\left(M_{1}^{N}\right)}_{21}\right] & =1\\ \to \alpha_{1}\beta_{1}\sin^{2}\left[N\Phi \right] & =\sin^{2}\Phi . \end{array}$$
  Now we turn to consider ${\left(M_{1}^{N}\right)}_{11}=0$ and ${\left(M_{1}^{N}\right)}_{22}=0$ , so
  (20)
  $$\begin{array}{cc} \sqrt{1-\alpha_{1}\beta_{1}}{\text{e}}^{\text{i}\phi_{1}}U_{N-1} & =U_{N-2}\\ \sqrt{1-\alpha_{1}\beta_{1}}{\text{e}}^{-\text{i}\phi_{1}}U_{N-1} & =U_{N-2}. \end{array}$$
  To satisfy above equations simultaneously, we find *ϕ*_1_ = 0. The transmission phase of the unit cell is null. With *ϕ*_1_ = 0, the corresponding Bloch phase would become
  (21)
  $$\cos\Phi =\sqrt{1-\alpha_{1}\beta_{1}}.$$
  Then, the last remaining condition to simultaneously meet ${\left(M_{1}^{N}\right)}_{11}=0$ and ${\left(M_{1}^{N}\right)}_{22}=0$ is
  (22)
  $$\begin{array}{ll} & \sqrt{1-\alpha_{1}\beta_{1}}U_{N-1}-U_{N-2}\\ & \;=\frac{\cos\Phi \sin\left[N\Phi \right]-\sin\left[N\Phi \right]\cos\Phi +\cos\left[N\Phi \right]\sin\Phi }{\sin\Phi }\\ & \;=\cos\left[N\Phi \right]\Rightarrow 0. \end{array}$$
  So we need to require cos[*N*Φ] = 0.

In short, if the unit cell is not operated at CPAL, to occur CPAL at a finite periodic PT-symmetric system, the corresponding conditions are *ϕ*_1_ = 0 and cos[*N*Φ] = 0.

## Notes

(9)
$$1-\frac{1}{T_{N}}=\left(1-\frac{1}{T_{1}}\right)\frac{\sin^{2}\left[N\Phi \right]}{\sin^{2}\Phi },$$

obtained from an extension of generalized unitary relation. Due to non-negative property of $\frac{\sin^{2}\left[N\Phi \right]}{\sin^{2}\Phi }\geq 0$ , when the unit cell is operated at the symmetry phase with *T*_1_ < 1, we can further obtain *T*_*N*_ ≤ 1. This result indicates that the scattering behaviors of any finite periodic systems made of the unit cells having symmetry phase would eventually have symmetry phase or exceptional point, for any *N*. Now, if the unit cell is operated at an exceptional point, it obeys *T*_1_ = 1. Again, with non-negative property of $\frac{\sin^{2}\left[N\Phi \right]}{\sin^{2}\Phi }\geq 0$ , we can obtain *T*_*N*_ = 1. Thus, any N-cell systems made by unit cells having an exceptional point would perform the exceptional point. On the other hand, for unit cells having broken symmetry phase *T*_1_ > 1, we can have *T*_*N*_ ≥ 1. As a result, any N-cell systems made of the unit cells at broken symmetry phase would have broken symmetry phase or exceptional point.
